# The effectiveness of multidisciplinary interventions based on health belief model on musculoskeletal pain in the elderly living in nursing homes: a study protocol for a randomized controlled trial

**DOI:** 10.1186/s13063-024-08243-1

**Published:** 2024-06-21

**Authors:** Sogand Habibi, Sedigheh Sadat Tavafian, Reza Maghbouli, Ali Montazeri

**Affiliations:** 1 Department of Health Education and Health Promotion, Faculty of Medical Sciences, Tarbiat Modares University, Tehran, Iran; 2 School of Medicine, Iran University of Medical Sciences, Tehran, Iran; 3 Population Health Research Group, Health Metrics Research Center, Iranian Institute for Health Sciences Research, ACECR, Tehran, Iran; 4 Faculty of Humanity Sciences, University of Science and Culture, ACECR, Tehran, Iran

**Keywords:** Musculoskeletal disorder, Elderly, Health belief model, Pain

## Abstract

**Background:**

Due to the burden of musculoskeletal diseases in the elderly and the multifactorial nature of such conditions, controlling the pain caused by these disorders requires multidisciplinary approach. This approach requires the participation of the elderly in applying effective prevention measures. This study aims to design a multidisciplinary educational intervention based on health belief model (HBM) for elderly residents of nursing homes.

**Methods:**

This is a parallel randomized clinical trial among elderly people aged 60 years and over living in a nursing home who suffer from musculoskeletal pain. Eligible participants will be divided into two groups including the intervention group who will receive a multidisciplinary intervention (vitamin D consumption, psycho-social stress management, and physiotherapy) and the control group who will receive usual care. Data collection instruments will include demographic data, the Depression, Anxiety, and Stress Scale (DASS), the visual analogue scale (VAS), and a self-designed questionnaire containing the HBM constructs. The interventions will be carried out by the educational team (general practitioner, psychologist, physiotherapist, and health education specialist). Interventions include changing the wrong beliefs of the elderly, taking 800 units of vitamin D daily, daily walking exercise by the elderly for at least 30 min and maintaining proper body posture during daily activities, muscle relaxation, relaxation techniques, regular exercise, examining their diet and eliminating stimulants (such as smoking and coffee), regular visits with friends and family, and deep breathing techniques. All questionnaires will be completed by the elderly before, after, 3, and 6 months after the intervention.

**Discussion:**

The present study will evaluate the effect of an educational intervention based on a multifaceted pain control approach for elderly people who reside in nursing homes in order to reduce musculoskeletal pain in the elderly living in nursing homes. One of the features of this study is its focus on improving the health of elderly residents in nursing homes. Given the increase in the elderly population worldwide, the findings from the current trial might benefit elderly populations.

**Trial registration:**

IRCT20220904055881N1. Registered on 11 February 2023.

**Supplementary Information:**

The online version contains supplementary material available at 10.1186/s13063-024-08243-1.

## Background

Musculoskeletal disorders (MSDs) include over 150 diseases, which are characterized by conditions ranging from fractures, sprains, and strains to lifelong disabilities and usually with pain (often chronic), limited mobility, skill, and overall performance levels. Osteoarthritis, rheumatoid arthritis, osteoporosis, and other diseases are among the most common of these disorders [1–3]. Approximately 1.71 billion people have MSDs worldwide [2].

In 2022, there were 771 million people aged 65 years or over globally, 3 times more than the size in 1980 (258 million). The older population is projected to reach 994 million by 2030 and 1.6 billion by 2050 [4]. Iran has been argued that this country had the highest increase in the burden of MSDs over the past three decades worldwide [5]. Behavioral factors such as inactivity and repetitive movements, as well as non-behavioral factors such as aging, gender, and genetics, are involved these disorders [6]. Pain is one of the most noticeable symptoms of these disorders. Studies show that the elderly suffer from musculoskeletal pain [7, 8]. Inactivity [7], stress and depression [9], and vitamin D deficiency [10–13] can be mentioned among the causes of MSDs.

Control of MSDs in the elderly is crucial because it usually leads to disability and a reduction in their quality of life [14–16]. There are different types of methods for reducing pain, such as nutrition (vitamin D intake), physiotherapy, exercise, and stress management [12, 17–20]. Despite the proven effectiveness of many pain-reducing methods, adherence to these methods in old people is an important challenging issue due to disease characteristics and poor individual perception.

One of the inevitable changes of old age is cognitive and perceptual changes, which have negative effects on the quality of life of the elderly. One of the perceptual changes in the elderly that influences inactivity is the fear of falling [21]. Low self-confidence and low self-efficacy [21], as well as their lower knowledge and awareness [22] about the benefits of physical activity, is a big obstacle among elderly people. Different chronic diseases in old age is another issue [23] that could be contributed to inactivity in this age group. Immobility and movement phobia caused by fear, lack of knowledge about the benefits of vitamin D consumption, low income, and inability to buy and cook healthy food are among the reasons for vitamin D deficiency in old people [24].

Controlling and changing wrong beliefs in the elderly is essential, and there are various models which have been used, especially with the aim of reducing MSDs [25].

Considering the role of the individual’s perception in performing preventive behaviors, the health belief model (HBM) has been widely used to modify wrong beliefs in the elderly in order to change their behavior. This model emphasizes the role of modifying factors (demographic, psychological, characteristics personality and structural) and individual’s perceptions together in determining the probability of performing the behavior, which can be a very powerful combination to change the behavior [26]. According to this model, the adoption of health behavior is effective when a person believes in the benefits/barriers of preventive behavior to decrease his/her disease and also believes that he/she is susceptible to the disease which may be so severe and threaten [27]. The individual’s perception can have a positive or negative effect on doing preventive behaviors [28–30]. The lower perception of the behavior barriers as well as the higher self-efficacy causes the more preventive behaviors performing against these disorders [29]. In various studies, the relationship between self-efficacy [31] and understanding the benefits of exercise in preventing osteoporosis in women and ultimately reducing pain has been reported [32]. Moreover, the relationship between promoting self-efficacy regarding preventive behaviors of osteoporosis [28] and reducing barriers in promoting physical activity [33] has been proven. HBM-based empowerment program can be an effective strategy for increasing self-perceived efficacy and benefits and also reduce perceived barriers in the elderly regarding preventive behaviors [28, 34].

Due to the multifaceted nature of the factors affecting musculoskeletal pain, multidisciplinary approach management of these pains could be effective. The previous evidences argued that the preventive influential factors in MSD control include stress management, physical activity, and vitamin D intake which should be targeted in their interventional program design, and ultimately, should be educated through educational intervention for older adults it be resulted in reducing pain. This approach requires the participation of the elderly in improving their wrong behaviors and choosing effective prevention methods.

### Objective

Since in this regard, according to our knowledge, there is no multidisciplinary educational program based on a suitable model for solving MSDs in Iranian elderly, this study aims to design an educational intervention based on the health belief model with the aim of changing wrong beliefs and trying to perform the target behaviors according to the effective factors in causing pain (stress, vitamin D deficiency, and physiotherapy) is designed to reduce musculoskeletal pain in the elderly living in nursing homes in Tehran, Iran.

### Trial design

**Fig. 1:**
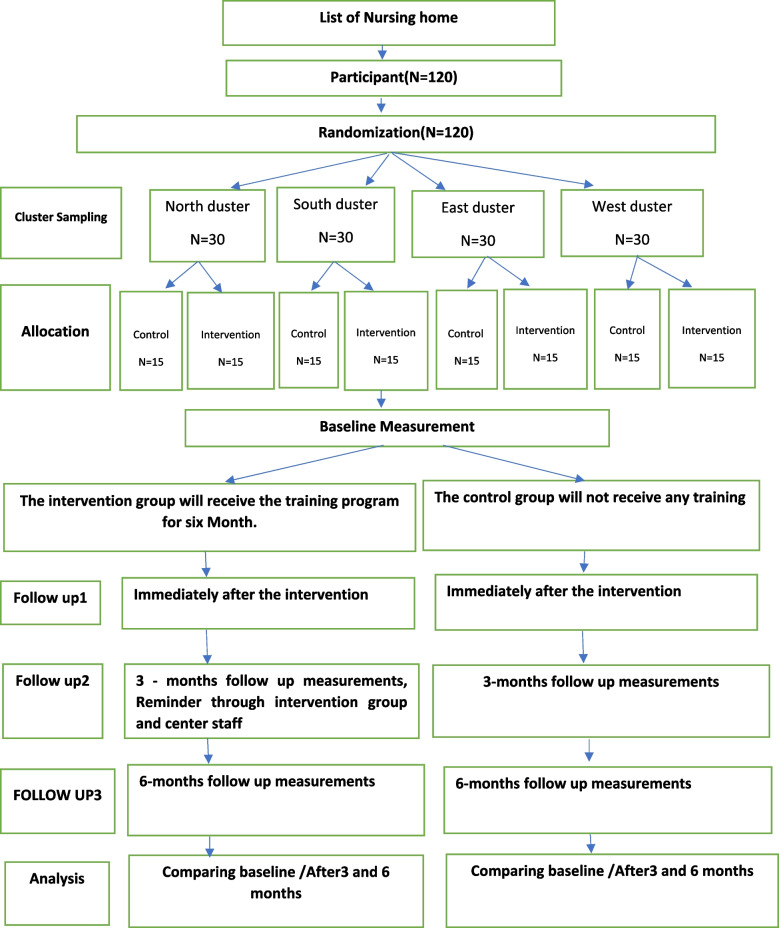
The flowchart of the randomized controlled protocol

**Fig. 2:**
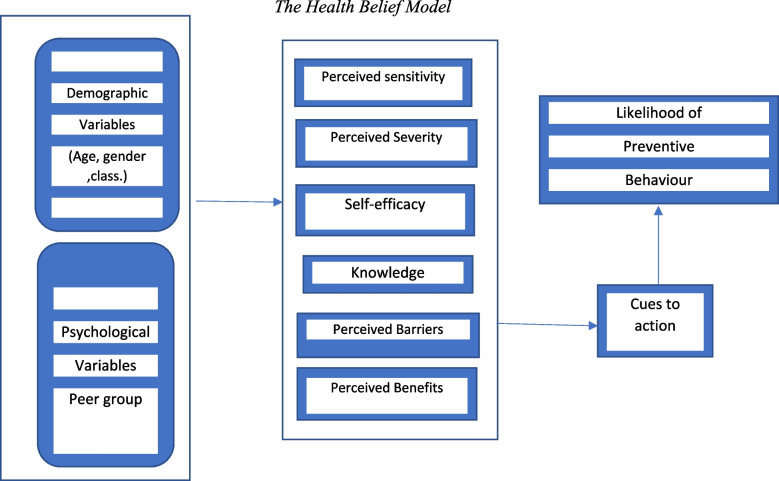
The interaction of construct model and individual characteristics in performing behavior

A parallel group randomized clinical trial will be conducted to assess the effectiveness of a health belief model based multidisciplinary intervention to reduce musculoskeletal pain in the elderly living in nursing homes. This protocol was developed and reported according to the recommendations of the Standard Protocol Items Recommendations for Interventional Trials (SPIRIT) [35], and will be conducted and reported following the Consolidated Standards of Reporting Trials (CONSORT) (Figs. 1, 2).

## Methods

This study will be carried out in two phase as follows:

1. Development and psychometric analysis of the study instrument

At this stage, a questionnaire will be designed based on the HBM constructs. Various statements will be included in the questionnaire based on previous studies on elderly musculoskeletal pain. The final instrument for analyzing the psychometric properties will be prepared (Table 1).

**Table 1:**
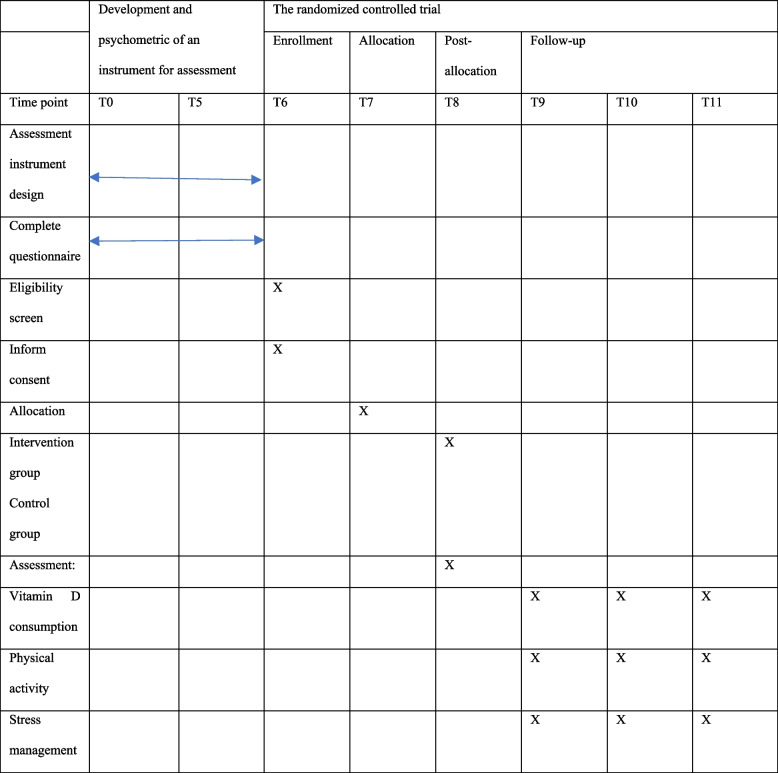
Schedule of enrolment, multidisciplinary intervention, and assessment of the intervention following the Standard Protocol Items Recommended for Clinical Trials (SPIRIT) guideline

2. Conducting a clinical trial

### Study setting

The study will be carried in a nursing home in Tehran, Iran. There is evidence that usually a sample of people living in Tehran would be a representative of Iranian population [36, 37].

### Eligibility criteria

The inclusion criteria for participation in the study are being aged 60 years and above, elderly people suffering from musculoskeletal pain, being resident in nursing homes, and being able to perform physical activities and respond to questions. Exclusion criteria include elderly people with Alzheimer’s, mobility impairments, and individuals who use medication for pain control.

### Who will take informed consent?

The necessary coordination will be done by the health education specialist with the relevant organizations. After obtaining permission from the nursing home, the health education specialist is responsible for explaining the objectives of the study to the nursing home officials and also obtaining informed consent from the elderly participating in the study.

### Intervention

This is a multifaceted intervention to reduce the pain of the elderly by the intervention team (Table 2). The intervention team includes a general practitioner, psychologist, physiotherapist, and health education specialist.

- Physical activity would contain daily walking exercise by the elderly for at least 30 min and maintaining proper body posture during daily activities. Walking is done on a flat, un-sloped surface in their nursing home for half an hour. The proper posture of the body means a combination of the position of the different joints of the body relative to each other at the same time. The position of each joint will affect the position of the other joints. The proper posture is the position in which the least pressure is placed on the joints and the muscle activity is at its lowest level [38]. The proper posture involves proper position of the joints while standing, walking, sitting, and sleeping that will be laid in least stressed (to support muscles, joints, and ligaments during movement and activity). Sessions are held for 8 weeks, with three 1-h sessions per week, to maintain proper posture [39]. The training is done by the physiotherapist in the nursing home. First, how to maintain the correct body position and how to do it by the physiotherapist will be described, then he asks the elderly to perform the correct movements under the supervision of the physiotherapist to prevent injury. Films and posters are used for training as well as reminders.
- Vitamin D intake: A session will be given by the doctor in the form of a lecture about the benefits of vitamin D consumption for an hour. The doctor is obliged to answer the questions of the elderly. Each elderly person is given a daily dose of 600 to 800 IU of vitamin D tablets [40] for 6 months. One of the employees of the nursing home will be responsible for giving pills to the elderly daily. The doctor is obliged to answer the questions of the elderly during the intervention period.
- Psychological sessions will be conducted by a psychologist. Considering the conditions and facilities available, stress and depression reduction sessions should be carried out in such a way that all elderly people can benefit from them. These sessions include examining the elderly’s sleep, muscle relaxation, relaxation techniques, regular exercise, examining their diet and eliminating stimulants (such as smoking and coffee), regular visits with friends and family, and deep breathing techniques. These sessions are held by the psychologist for 8 weeks with 2 sessions per week, and each session lasts 90 min [41]. Training sessions in the nursing home will be conducted in the form of group discussions and proper training of techniques.
- Health belief model educational sessions will be held for changing the perceptions and beliefs of the elderly. After completing the self-designed questionnaire, training sessions are determined for each constructs. Training is done according to the type of constructs. Training sessions include group discussion, use of role models, increasing self-efficacy, etc. Meetings are held in the nursing home for 1 h, if the elderly wish, the meeting time can be extended. Considering that the model constructs target intervention behaviors in addition to the belief of the elderly, the presence of a health education specialist in other meetings is necessary.

**Table 2:** Multidisciplinary intervention to reduce musculoskeletal pain based on the health belief model

Item	Intervention
Educational content	A series of videos and posters to teach proper body posture during daily activities, stress control and how to take vitamin D
Time line	Sessions are held for 8 weeks, with three 1-h sessions per week, to maintain proper posture. 8 weeks with 2 sessions per week, to control stress
Component	Health belief model constructs aimed at reducing perceived sensitivity, perceived severity and perceived barriers, increasing self-efficacy and perceived benefits and knowledge
Methods	Based on the constructs of the HBM model, various methods such as lectures, group discussions are used
Responsibilities	All team members are present during the intervention period. After the end of the intervention period, one of the staff members of each center will check the steps of the interventions and the adherence of the elderly to the interventions and send the relevant reports to the health education expert on a weekly basis. The team doctor and health education specialist will periodically meet with the elderly

### Strategies to improve adherence to interventions

As a reminder, one of the staff of the nursing home communicated with the intervention group and reminded them to do the interventions regularly. The health education specialist periodically monitors the implementation and maintenance of the interventions after the completion of the interventions. Finally, a reminder training will be provided for the intervention group after 3 months. The control group will not receive any intervention, but a training package will be provided to them after the study ends.

### Provisions for post-trial care

Considering the vulnerability of the target group, all health aspects of the plan are considered in all stages and possible complications are evaluated. The presence of a doctor in all meetings is necessary. Moreover, all participants are asked to report any possible side effects to be assessed by physician.

### Outcomes

The primary outcome of this study is to reduce musculoskeletal pain in the elderly. The secondary outcomes would be reduction in psychological distress (depression, anxiety, and stress) and improvements in beliefs among elderly using the health belief model constructs.

### Sample size

With a confidence level of 95% and a test power of 80%, the number of samples required for each group (intervention and control) is determined based on study conducted by Rostami et al. [42]. The required number of samples is estimated 55 per each group. Considering a 10% drop out, a sample of 60 participants per each group will be included.

### Recruitment

The necessary coordination will be done by the health education specialist with the relevant organizations. After obtaining permission from the nursing home, the health education specialist is responsible for explaining the objectives of the study to the nursing home officials and also obtaining informed consent from the elderly participating in the study. The intervention team works in cooperation with the main manager of the nursing home. Interventions will be carried out according to the program. All team members are present during the intervention period. After the end of the intervention time, one of the employees of each center will check the steps of the interventions and the adherence of the elderly to the interventions and send the relevant reports to the health education specialist on a weekly basis. The team doctor and health education specialist (principal investigator) will periodically meet with the elderly. Nursing homes that agree to participate in the project are included in the study. Potentially eligible seniors are enrolled in the study. In this study, the intervention team performs the training. The control group will receive the delayed intervention after completing the 6-month follow-up questionnaire.

### Sampling

For sampling in the first stage, Tehran will be divided into four clusters of north, south, east, and west. A lottery method will be used to select nursing homes in each cluster, by assigning a number to each nursing home. The numbers will be written on a card, which will be placed in a container and thoroughly mixed. Two cards are then selected, one card representing the intervention group and the other representing the control group. The intervention and control groups (each consisting of 15 individuals) are selected from elderly homes according to the criteria for identification and selection. The reason for sampling from all four clusters is to generalize the results to the entire population.

### Blinding

This study is not blinded. The main investigator is a health education specialist who manages the data. Allocation of people to intervention and control groups will be done randomly (lottery).

### Outcome measures

1. A visual analogue scale (VAS) for measuring pain [43]. A pain scale is a tool that doctors use to help assess a patient’s pain level. A person usually self-reports pain using a specially designed scale, sometimes with the help of a doctor, parent, or guardian. The pain scale may be used on admission to the hospital, during a doctor’s visit, during physical activity, or after surgery. Visual analogue scale or VAS is the pain ruler that includes a horizontal line that is graded from 0 to 10, where 0 indicates absolute painlessness and 10 indicates unbearable pain. At one end is “no pain” and at the other end is “the worst pain” or “indescribable pain.” The person puts a cross X on the line to indicate the intensity of their pain. A doctor then measures the line with a ruler to obtain a pain score.
2. The Depression, Anxiety, and Stress Scale (DASS-21) will be is used to measure depression and stress. The questionnaire contains 21 items (7 items per each component). The depression subscale includes phrases that measure unhappy mood, lack of self-confidence, hopelessness, worthlessness of life, lack of interest in involvement in affairs, lack of enjoyment of life, lack of energy, and empowerment. The anxiety subscale has expressions that attempt to assess physiological hyperarousal, fears, and situational anxieties, and the stress subscale includes terms such as difficulty in achieving relaxation, nervous tension, irritability, and restlessness [44].
3. The HBM questionnaire: as indicated earlier a self-designed questionnaire specifically will be developed for the study including items covering knowledge, perceived severity, perceived sensitivity, perceived benefit, perceived barriers, self-efficacy, and cues to action. Various statements will be included in the questionnaire based on previous studies on elderly musculoskeletal pain. The psychometric characteristics of the questionnaire, including quantitative and qualitative face validity, quantitative and qualitative content validity, construct validity, and reliability, will be assessed. To assess the validity of the statements, 10 elderly people will be involved, and the formal validity of the statements will be done. To determine the content validity ratio (CVR), the index designed by Lawshe [45] is used and to calculate the content validity index (CVI) the method of Bausell Waltz is used [46]. The questionnaire will be given to 15 specialists (10 health education specialists, 2 physiotherapists, 2 psychologists, and 1 general practitioner) to measure the CVI and CVR. Quantitative and qualitative face validity of the article will be done. Exploratory factor analysis will be used to assess the construct validity. In order to determine Cronbach’s alpha coefficient, a questionnaire is given to 30 elderly people with inclusion and exclusion criteria. Afterwards, we use construct validity to confirm the dimensions of the tools and items, during which path analysis and exploratory factor analysis are used. After exploratory factor analysis, dimensions and items would be determined.
4. In addition, demographic data including age, sex, education, BMI, and history of disease will be collected.
5. The behavior questionnaire, which includes three behaviors (vitamin D consumption, physical activity, and stress management), filled out by the elderly before and after the intervention.

Data will be collected using the study measures. The outcomes will be assessed in four points in time: before, immediately, 3, and 6 months after the interventions. Questionnaires of behavior and health belief model are measured with a 5-point Likert scale from completely disagree to completely agree.

### Data management

Participants’ personal information is assigned a unique identification code, stored in a password-protected format, and accessible only to the principal investigator. Participants cannot be identified in manuscripts, reports, or research-related presentations. Data that will be entered into SPSS will be double checked to promote data quality.

### Data analysis

To conduct statistical analysis of data, the IBM SPSS Modeler version 21 will be used. Descriptive statistics such as frequency and percentage, mean, and standard deviation will be used. For classification variables, chi-square and Fisher’s exact tests will be used to determine the difference between groups. Before and after evaluation, intra-group comparisons are determined by paired *t*-test. Independent group means will be evaluated with a 95% confidence interval. The Kolmogorov-Smirnov test will be used to check the normality of data. Finally, after adjusting for gender and age group, a multivariable linear regression model will be used to examine the combined effects of vitamin physiotherapy and social-psychological interventions on changes in pain scores. Repeated measure analysis of variance will also be used. This study will use exploratory factor analysis to identify the underlying categories of an item set.

### Dissemination policy

Trial results will be submitted to peer-reviewed journals for publication. Part of the data, such as information regarding the main outcome, can be shared.

## Discussion

This study aims to design a multidisciplinary educational intervention based on health belief model (HBM) for elderly residents of nursing home. One of the features of this study is its focus on improving the health of elderly residents in nursing homes. Considering that elderly suffer from various diseases such as musculoskeletal pain, the main purpose of this study was to design a multimodal intervention based on HBM to obtain preventive behaviors of musculoskeletal pain in the elderly. The positive effect of interventions on teachers [47] as well as combined programs (supplement consumption and physical activity) based on HBM [48] has been proven. Various studies have also confirmed the positive effect of HBM-based interventions [49, 50] and the effect of multimodal interventions on reducing pain in the elderly [51]. The current study might provide similar findings. Different programs have been designed in various countries to reduce musculoskeletal pain, such as the multidisciplinary rehabilitation programs in Germany that aim to improve the performance and workability of individuals [52, 53]. In the USA, the National Institute of Health has allocated a budget for musculoskeletal conditions [3]. Routine care is provided for the elderly in healthcare centers in Iran, but there is no specific care for preventing and controlling musculoskeletal disorders, and in elderly care centers may not be provided due to lack of compliance with health standards [54]. Even in the interventional researches that are done on the elderly population, the elderly group living in the nursing home is eliminated [55]. Also, in most research related to physical activity, the elderly is left out due to age-related diseases [56]. Therefore, the strengths of this study we can mention the combined intervention to reduce musculoskeletal pain in the elderly living in nursing homes and the group interaction of the elderly in the intervention group, which reduces stress and follows the educational program. Also, sampling from several centers and the presence of a control group that only receive routine interventions are other strengths of this study, because the elderly care status can be improved if the results of this study will be positive.

### Limitations

Among the limitations of this study is the inability to control the conditions of the nursing home, and the elderly may have difficulty interpreting their pain when using the VAS scale. Self-reporting of information is another limitation of this study.

### Supplementary Information

Additional file 1. SPIRIT checklist. 

## Data Availability

Non-applicable participant level data will not be shared.

## Abbreviations

MSDs: Musculoskeletal disorders
HBM: Health belief model
DASS: Depression, Anxiety, and Stress Scale
VAS: Visual analogue scale
CVI: Content validity index
CVR: Content validity ratio
