# Correction: The effectiveness of multidisciplinary interventions based on health belief model on musculoskeletal pain in the elderly living in nursing homes: a study protocol for a randomized controlled trial

**DOI:** 10.1186/s13063-024-08325-0

**Published:** 2024-07-18

**Authors:** Sogand Habibi, Sedigheh Sadat Tavafan, Reza Maghbouli, Ali Montazeri

**Affiliations:** 1 Department of Health Education and Health Promotion, Faculty of Medical Sciences, Tarbiat Modares University, Tehran, Iran; 2 School of Medicine, Iran University of Medical Sciences, Tehran, Iran; 3 Population Health Research Group, Health Metrics Research Center, Iranian Institute for Health Sciences Research, ACECR, Tehran, Iran; 4 Faculty of Humanity Sciences, University of Science and Culture, ACECR, Tehran, Iran

**Correction: Trials 25, 406 (2024)**

**https://doi.org/10.1186/s13063-024-08243-1**

Following the publication of the original article [1], we were notified of a typo in the first name of the last author.

Originally published name: Ail Montazeri

Corrected name: Ali Montazeri

The original article was corrected.
